# Effectiveness of physical activity interventions for overweight and obesity during pregnancy: a systematic review of the content of behaviour change interventions

**DOI:** 10.1186/s12966-019-0859-5

**Published:** 2019-11-01

**Authors:** Caragh Flannery, Milou Fredrix, Ellinor K. Olander, Fionnuala M. McAuliffe, Molly Byrne, Patricia M. Kearney

**Affiliations:** 1 School of Public Health, University College Cork, Cork, Ireland; 2 Health Behaviour Change Research Group, School of Psychology, National University of Ireland, Galway, Ireland; 3 Centre for Maternal and Child Health Research, School of Health Sciences, City, University of London, London, United Kingdom; 4 Perinatal Research Centre, School of Medicine, University College Dublin, National Maternity Hospital, Dublin, Ireland

**Keywords:** Physical activity, Pregnancy, BMI, Intervention, Behaviour change, Behaviour change techniques, Systematic review

## Abstract

**Background:**

Behaviour change techniques (BCTs) employed within PA intervention for pregnant women with a healthy body mass index (BMI) have been previously identified, however, these BCTS may differ for other weight profiles during pregnancy. The aim of this current review was to identify and summarise the evidence for effectiveness of PA interventions on PA levels for pregnant women with overweight and obesity, with an emphasis on the BCTs employed.

**Methods:**

A systematic review and meta-analysis of PA intervention studies using the PRISMA statement was conducted. Searches were conducted of eight databases in January 2019. Strict inclusion/exclusion criteria were employed. The validity of each included study was assessed using the Cochrane Collaboration’s tool for assessing risk of bias. The primary outcome measure was change in PA levels, subjectively or objectively measured, with physical fitness as a secondary outcome. All intervention descriptions were double coded by two authors using Michie’s et al’s BCT taxonomy V1. Meta-analyses using random effect models assessed the intervention effects on PA. Other PA outcomes were summarised in a narrative synthesis.

**Results:**

From 8389 studies, 19 met the inclusion criteria 13 of which were suitable for inclusion in a meta-analysis. The remaining 6 studies were described narratively due to insufficient data and different outcome measures reported. In the meta-analysis, comparing interventions to a control group, significant increases were found in the intervention group for metabolic equivalent (SMD 0.39 [0.14, 0.64], Z = 3.08 *P* = 0.002) and physical fitness (VO_2_ max) (SMD 0.55 [0.34, 0.75], Z = 5.20 *P* = < 0.001). Of the other six, five studies reported an increase in PA for the intervention group versus the control with the other study reporting a significant decrease for women in their 3rd trimester (*p* = 0.002). ‘Self-monitoring of behaviour’ was the most frequently used BCTs (76.5%), with ‘social support’ being newly identified for this pregnant population with overweight or obesity.

**Conclusions:**

This review identified a slight increase in PA for pregnant women with overweight and obesity participating in interventions. However, due to the high risk of bias of the included studies, the results should be interpreted with caution. PA measures should be carefully selected so that studies can be meaningfully compared and standardised taxonomies should be used so that BCTs can be accurately assessed.

## Background

Overweight and obesity during pregnancy is an increasing public health concern. Overweight is defined as BMI ≥25 kg/m^2^ and obesity is defined as a BMI ≥30 kg/m^2^ which is assessed at the first antenatal consultation [1]. Overweight and obesity is associated with a number of adverse maternal and neonatal outcomes including increased rates of gestational diabetes mellitus (GDM), pre-eclampsia, caesarean section, instrumental delivery and preterm delivery [2, 3]. Additionally excessive gestational weight gain is associated with weight retention and type 2 diabetes in the longer term [4, 5].

Physical activity has been identified as a modifiable lifestyle factor that could help prevent pregnancy complications, help with weight management and reduce the risk of GDM for women with overweight and obesity [6]. Previous research has found that physically active pregnant women report better health than less physically active women as well as an increase in functional ability and a reduction in nausea, fatigue and stress [7–9]. Despite the significant health benefits, based on self-report, women tend to be less active in pregnancy due to fatigue and discomfort [10, 11]. International guidelines recommend 30 min of daily moderate intensity physical activity for pregnant women [12–15]. A review which updated the latest evidence concerning exercise during pregnancy found that in the United States only 15.8% of women engaged in exercise during pregnancy [16]. Similarly, low levels of physical activity have been reported in an Irish cohort of pregnant woman with only 21.5% of women meeting the current recommendations [9, 11]. Furthermore, a study examining lifestyle changes using the Pregnancy Risk Assessment Monitoring system (PRAMS) in Ireland found that adherence to physical activity guidelines of moderate intensity activity was low (12.3%) but was particularly low for pregnant women with overweight and obesity (6.4%) [17]. Therefore, pregnant women with overweight and obesity should be encouraged to follow an exercise programme in order to get the best health outcomes for both mother and baby [18].

Behavioural change is complex and involves identifying effective and efficient techniques to bring about change [19]. These techniques are called behaviour change techniques (BCTs) and are defined as ‘an active component of an intervention designed to change behaviour’ pg. 145 [20]. In order to identify the intervention content or behavioural component of an intervention, the BCT taxonomy V1 was developed [20]. The BCT Taxonomy V1 consisting of 93 different BCTs (16 categories) is a useful tool to extract the active components of successful and unsuccessful behaviour change interventions.

However, reviews of lifestyle interventions during pregnancy are varied and results to date are conflicting [21–23]. Many of the interventions promoting lifestyle changes throughout pregnancy are multidimensional incorporating a combination of diet and physical activity [2, 22, 24, 25]. These interventions tend to focus on medical or obstetric outcomes such as reducing excessive gestational weight gain (GWG) or GDM with less focus on the behavioural outcomes such as physical activity.

According to a review by Currie et al. (2013) which evaluated the content of physical activity interventions in pregnancy, interventions within the review were most effective when BCTs were employed and delivered face to face [26]. However, there is uncertainty around which underlying BCTs are most effective. Collins et al. suggested two components that need to be explored in order to identify effective interventions. These are intervention programme (employed BCTs) and intervention delivery (intervention provider, format, setting, recipient, intensity, duration and fidelity of the intervention) [27]. A review examining behaviour-change interventions for obese adults with additional risk factors or co-morbidities found suggestive evidence for an association between greater numbers of BCTs and greater weight loss [28]. Furthermore, a review examining intervention features of dietary and physical activity interventions for patients with type 2 diabetes revealed BCTs associated with clinically significant reductions in HbA_1c_ [29]. Previous systematic reviews in the area of pregnancy [26, 30] have assessed intervention effectiveness including GWG [21–23, 31, 32] and GDM [33] but have not examined the intervention programme content itself.

BCTs have been retrospectively identified in a number of systematic reviews [24, 34]. The identification of optimal BCTs necessary for increasing physical activity in a healthy adult population found six important techniques including: providing information on the likely consequences of specific behaviour, action planning, reinforcing effort or progress, providing instructions, facilitative social comparison and time management [24]. However, the techniques associated with increasing physical activity for adults with obesity were different, using BCTs such as ‘teach to use prompts/cues’, ‘prompt practice’ or ‘prompt rewards’ instead. Thus, to develop effective physical activity interventions it may be important to consider tailoring intervention techniques to the target population [35]. The significance of BCTs may be different for pregnant women compared to non-pregnant women since pregnancy is a unique time where women may be more receptive to improving health behaviours [36]. In pregnancy, using the most up-to-date BCT taxonomy, Currie et al. identified the most common BCTs for healthy weight pregnant women, including ‘goal setting’, ‘feedback and planning’, ‘repetition and substitution’, ‘shaping knowledge’ and ‘comparison of behaviours’ [26]. Furthermore, the value of these techniques is likely to depend on the weight profile of the pregnant population and successful BCTs may differ for pregnant women with overweight and obesity compared to pregnant women with a healthy BMI [37–40].

Therefore, the aims of this systematic review and meta-analysis was to identify and summarise the evidence for the effectiveness of physical activity interventions for pregnant women with overweight and obesity on physical activity levels and identify which BCTs were most frequently used in these interventions and determine which were most effective in improving physical activity levels.

## Methods

This systematic review and meta-analysis were reported in accordance with the Preferred Reporting Items for Systematic reviews and Meta-Analyses (PRISMA) statement [41]. The review protocol was pre-registered with the International Prospective Register of Systematic Reviews (PROSPERO) database (CRD42016033423).

### Eligibility criteria

#### Types of studies

Eligible study designs included pilot randomised controlled trials, randomised control trials (RCTs), non-randomised control trials, quasi RCTs, and quasi-experimental studies of physical activity interventions, aimed at maintaining or increasing physical activity levels conducted in any setting. Furthermore, for inclusion, all interventions had to target pregnant women with overweight and obesity with a body mass index (BMI) ≥25 kg/m^2^, have at least one component focusing explicitly on physical activity, and include a discernible BCT in the intervention description. Control groups were classified as a comparator intervention or usual care if stated. Usual care would indicate standard antenatal care for pregnant women. Studies were included regardless of treatment intensity, duration or mode of delivery of the intervention. Only studies published in English were included. Studies published in the grey literature (non-peer reviewed or without scientific credibility) were excluded.

#### Types of participants

Participants included pregnant women with a pre-pregnancy or early pregnancy BMI ≥25 kg/m^2^ and singleton pregnancies.

#### Types of outcome(s) measures

Studies were included that reported any of the following primary outcome measures: change in physical activity levels subjectively (e.g., self-report) or objectively measured (e.g., step count) at baseline and post intervention. Secondary outcome included studies that reported VO_2_ max as a measure of physical fitness.

### Information sources

#### Searches

MEDLINE, EMBASE, PsychInfo, CINAHL, Cochrane Library, PEDro, SportDiscus and PubMed databases were searched from inception. The searches were undertaken in January 2019**.** The search strategy for each database is available in Additional file 1. Phrases and MESH headings for each component of the population, intervention, comparator and outcome framework (PICO), were combined using OR and then using AND (maternal, pregnancy, pregnant woman, expectant mothers; lifestyle, lifestyle modification, health promotion, behaviour change, physical activity, exercise, fitness, activities of daily living, human activities, group exercise, randomised controlled trial, intervention trials and clinical trials; standard care; physical activity, gestational weight gain and gestational diabetes). Manual searches of reference lists were conducted on all eligible articles following screening.

### Study selection

**Fig. 1:**
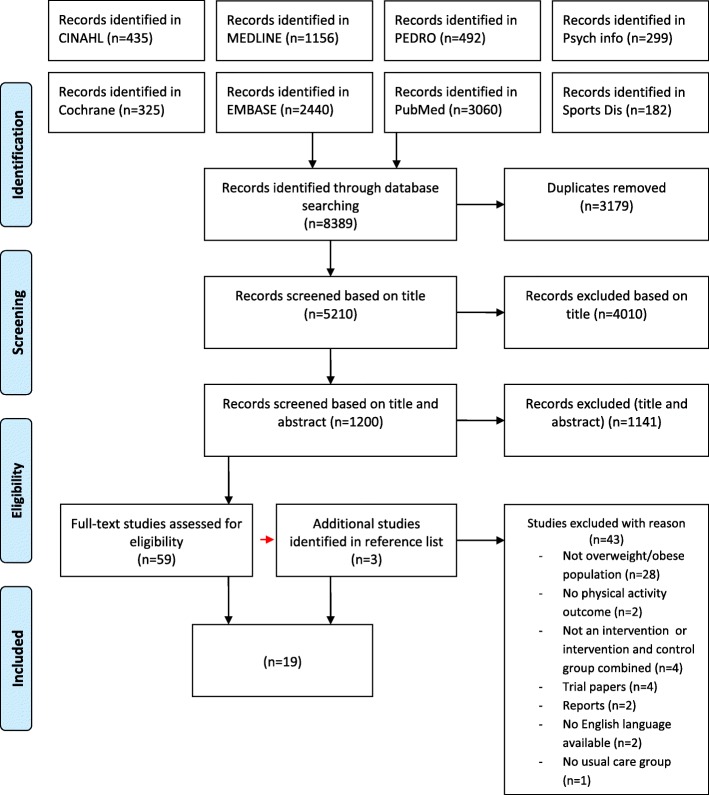
PRISMA Flow Diagram

One author (CF) conducted the searches and imported citations in to a reference management software package (Endnote version 7). Duplicates were removed. In the first screening stage, all titles of the search results were examined and irrelevant titles were removed if they did not meet the inclusion criteria. In the second stage, title and abstracts were screened. Ten percent of title and abstracts were double screened by authors (MB, EO, PK and FMA). Any discrepancies were resolved by consensus. Cohen’s kappa (*k)* was calculated to determine the extent of interrater agreement [42, 43]. In the third stage of the screening process, relevant articles were obtained in full and assessed against the inclusion and study quality criteria. Full text screening was conducted by (CF) and checks were made by 2 s reviewers (MB and PK); discrepancies were resolved by consensus. The number of articles at each stage can be seen in the PRISMA flow chart (Fig. 1).

### Data extraction

A data form was developed based on the Workgroup for Intervention Development and Evaluation Research (WIDER) framework for the scientific reporting of behaviour change interventions [44]. Data from each included study were extracted by one reviewer (CF) and independently checked by two others (MB and PK). In case of discrepancies, consensus was reached through discussion. Extracted data included detailed description of the interventions (study design, participant information, details of the intervention, sample size, type of contact and setting) and BCTs included in the intervention. Physical activity measures for baseline, pre and post intervention, where possible, were extracted from studies or calculated using reported means, standard deviations, and sample sizes at baseline, post-intervention.

### Coding of BCTs

The BCT taxonomy V1 was used to identify the behavioural components of the intervention within each included study. This validated taxonomy consists of 93 different BCTs divided into 16 categories. A BCT was only coded when it was explicitly mentioned in the intervention or supporting materials (study protocols). The BCT coding was completed independently by two reviewers (CF and MF) who underwent training in BCT coding using the BCT taxonomy. Inter-rater reliability was calculated [43] and discrepancies were discussed until 100% agreement was achieved.

#### Risk of bias assessment

Following the intensive screening process only RCTs were included, therefore, the validity of each included study was assessed using the Cochrane Collaboration’s tool for assessing risk of bias [45]. This tool assesses key methodological domains; sequence generation, allocation concealment, blinding of participants, personnel and outcome assessors, incomplete outcome data, selective outcome reporting, other sources of bias [45]. The risk of bias was assessed by one reviewer (CF) and in the case of uncertainty consensus was reached through discussion with two authors (MB and PK).

### Strategy for data synthesis

#### Effect of the intervention

Results from the included studies were combined in a meta-analysis if sufficient outcome data were available from at least two studies. When an intervention reported data at several time points during pregnancy, the last measure before birth was used. Continuous data were summarized as mean difference and standard deviations (SD). Where possible, means and SD were calculated from median and interquartile range [46]. Within the meta-analysis, primary and secondary physical activity outcomes reported on the same scale (e.g. MET, Steps and VO_2_ max) were combined using standardised mean differences (SMD). For all effect sizes, 95% Confidence Intervals (CI) were used and results were pooled using a random effects model (inverse-variance approach based on weighted SMDs) using Review Manager Software (version 5.3: Review Manger). Furthermore, the I^2^ statistic was used to indicate the percentage of total variation [45]. If data was not available for pooling outcomes, all other physical activity outcomes measures were combined in a narrative synthesis. To test the robustness of the findings, risk of publication bias was conducted using Stata (version 13.1). Funnel plots were generated and a test for statistical significance for funnel plot asymmetry was performed using Eggers test [47].

#### BCTs

A BCT was only coded when there was clear evidence of its inclusion in the intervention and it was identified as present by both reviewers. The total number of BCTs was recorded and the frequency of identified BCTs was quantified. Subgroup analysis was selected as a method to examine the effectiveness of different BCTs on outcomes included in the meta-analysis. Subgroup analysis would only be conducted if a meta-analysis was conducted with 10 or more studies. Pearson’s r correlation coefficient was used to investigate the relationship between the number of BCTs used and the outcome effect sizes.

## Results

### Study selection

Searches conducted in January 2019 found 8389 studies. Nineteen studies were included (Fig. 1), describing 3 pilot randomised controlled trials [48–50] and 16 randomised controlled trials [51–66] of which 2 were multicentre [60, 61], 2 were prospective [62, 63], 2 were parallel [64, 65] and 1 was a nested randomised controlled trial [66]. Cohen’s kappa (*k)* was calculated to determine the extent of inter-rater agreement during the screening phase and a substantial agreement was reached (k = 0.63). The total number of participants included in all studies was 7822, ranging from 12 [56] to 1924 [60] in individual studies.

Health outcomes measured in the interventions included gestational weight gain, fasting insulin, fasting glucose, gestational diabetes, gestational age (weeks), and infant birth weight (kg). Eight studies were investigations targeting physical activity promotion alone [48, 50, 53, 54, 56, 57, 64, 65] while 11 others were of interventions targeting diet and physical activity [49, 51, 52, 55, 58–63, 66]. Fourteen studies described their control groups as receiving standard routine antenatal care. There was no clear definition of standard antenatal care in these studies. Five studies described their control group as those who were not provided with the intervention [64], those who were not provided with physical activity recommendations or restricted from physical activity participation [50, 55]. The final two studies compared the intervention with a stretching group which included relaxation (respiratory exercises and light stretching) [57] or having access to additional information from a website [59].

### Characteristics of included studies

Studies were conducted in Australia [48, 56, 60, 66], the Netherlands [54], the United States of America (USA) [49, 50, 59], Brazil [53, 57], New Zealand [64], Ireland [58], the United Kingdom (UK) [61], Italy [63], Finland [52], Denmark [55, 62], Belgium [51] and Norway [65]. Twelve studies were interventions that targeted pregnant women with overweight and obesity [49, 50, 53, 54, 57–60, 63–66] while seven studies focused on pregnant women with obesity only [48, 51, 52, 55, 56, 61, 62] (See Table 1).

**Table 1:** Characteristics of included studies

Author & Year	Country	Study design	N	Age	BMI	Gestation	Pregnancy type	Other risk factors	Intervention detail (brief description, comparison)	Type of PA measure	PA outcome measure
Callaway et al 2010 [48]	Australia	Pilot RCT	50	Aged 18–45	BMI ≥ 30	Not specified	Not specified	Not specified	Intervention group: individualized exercise program with an energy expenditure EE goal of 900 kcal/ week *Comparison:* routine obstetric care	Self-report	Pregnancy Physical Activity Questionnaire (PPAQ) - MET (hr/week)
Oostdam et al 2012 [54]	Amsterdam	RCT	101	Not specified	BMI ≥ 25 or ≥ 30	Not specified	Not specified	At least one: macrosomia, history of GDM or relative with T2D	Exercise programme consisting of aerobic + strength exercises aimed top control blood glucose levels. *Comparison:* received normal care from obstetricians and or midwives	Objective	ActiTrainer accelerometer ActiGraph accelerometer - Total minutes per week of PA + MET cut -off values
Nascimento et al 2011 [53]	Brazil	RCT	82	Not specified	BMI 26–29	14–24 weeks	Not specified	Not specified	Two components: The exercise protocol consisting of light-intensity to moderate-intensity exercises + home exercise counselling. *Comparison:* no physical activity counselling, received routine prenatal care	Self-report	Women recorded the type + minutes of exercise in an exercise journal
Kong et al 2014 [50]	USA	Pilot RCT	37	Aged 18–45	BMI > 25 or > 30	Not specified	Singleton	Non-smoker, no prior history of chronic disease	Unsupervised walking program - Walking (150 min/week of moderate PA during pregnancy). *Comparison:* no physical activity recommendations, no restrictions from physical activity participation	Objective	StepWatch Activity Monitor (SAM) accelerometer - using step data (counts)
Seneviratne et al 2016 [64]	Auckland New Zealand	Two arm parallel RCT	75	Aged 18–40	BMI ≥ 25	< 20 weeks	Singleton	Not specified	Structured home-based exercise programme using magnetic stationary bicycles. *Comparison:* no intervention or heart rate monitor	Objective	Heart rate monitor - duration and intensity of cycling
Ong et al 2009 [56]	Western Australia	RCT	12	Aged 30 (±4 years)	BMI ≥ 30	Not specified	Singleton	Sedentary women, a normal 18 week scan	Home-based supervised exercise using an upright stationary cycle ergometer that each participant kept in their home during the intervention. *Comparison:* continued with their usual daily activities while receiving regular antenatal care	Objective and self-report	Aerobic Power Index sub maximum test and Pregnancy PA questionnaire
Santos et al 2005 [57]	Brazil	RCT	72	Aged ≥ 20	BMI ≥ 25	Not specified	Not specified	Non-smoking	Supervised PA consisting of warm up, heart rate monitored activity, upper and lower limbs, stretching and relaxation. *Comparison:* participated in once weekly sessions that included relaxation (respiratory exercises and light stretching (no aerobic or weight resistance) Participates were neither encouraged nor discouraged to exercise	Objective and self-report	Physical activity questionnaire) and the Aerobic Power Index sub maximum test- Vo2max
Garnaes et al 2016 [65]	Norway	Single centre, parallel group RCT	91	Aged ≥ 18	BMI ≥ 28	< 18 weeks	Singleton	Live fetus at 11–14 week ultrasound scan	Supervised exercise consisting of treadmill walking/jogging for 35 min (endurance) and resistance training for large muscle groups and the pelvic floor muscles. *Comparison:* ordinary maternity care by their midwife, GP and or obstetrician	Self-report	PA questionnaire - Frequency, duration and intensity of weekly PA
Dodd et al 2014 [60]	South Australia	Multicentre RCT	1924	Not specified	BMI ≥ 25	Between 10 and 20 weeks	Singleton	Not specified	Lifestyle Advice consisted of dietary + lifestyle intervention including dietary, PA and behavioural strategies + goal setting. *Comparison:* continued pregnancy care according to local hospital guidelines	Self-report	Health-enhancing PA (SQUASH) - MET (min/week)
Guelinckx et al 2009 [51]	Belgium	RCT	122	Not specified	BMI > 29	< 15 weeks	Not specified	White	Passive group: brochure consisting of diet and PA advice + tips to limit weight gain. Active group: received the same brochure and was actively counselled. Techniques of behavioural modification were used. *Comparison:* routine perinatal care	Self-report	Baecke questionnaire - Total score for PA from a minimum of 3 to a maximum of 15
Hawkins et al 2015 [49]	Western Massachusetts	Pilot RCT	68	Aged 18–40	BMI ≥ 25	< 18 weeks	Not specified	Hispanic women, participating in < 30 min PA per week	Achieve PA guidelines through increasing walking and developing a more active lifestyle. Dietary component: decrease foods high in saturated fat and increase fibre. *Comparison:* standard care	Self-report	Pregnancy PA Questionnaire (PPAQ) - average MET (h/week)
^a^Koivusalo et al 2016 [52]	Finland	RCT	269	Aged ≥ 18	BMI ≥ 30	< 20 weeks	Not specified	History of GDM	Dietary and PA counselling (minimum of 30 min of moderate intensity exercise and to adopt an overall active lifestyle). *Comparison:* received general antenatal care, information leaflets provided by the local antenatal clinics.	Self-report	Food frequency and PA questionnaire - Self report time spent weekly on PA
Poston et al 2015 [61]	UK	Multicentre RCT	1555	Aged > 16	BMI ≥ 30	Between 15 and 18 weeks (+ 6 days)	Singleton	Not specified	SMART goals, advice on self-monitoring, problem solving. Handbook about the intervention, theory and recommended food and PA. DVD of an exercise regimen. *Comparison:* routine antenatal appointments at their trial centre in accordance with local practice	Self-report	PA questionnaire (IPAQ) - MET (min/week)
Renault et al 2014 [62]	Copenhagen	Prospective RCT	389	Aged > 18	BMI ≥ 30	Between 11 and 14 weeks	Singleton	Read and speak Danish	Two intervention groups: (PA plus D and PA only) individually advised and encouraged to increase PA aiming at a daily step count of 11,000 steps. The diet intervention consisted of contact with an experienced dietician. *Comparison:* received usual hospital standard regimen for obese pregnant women	Objective	Pedometer - Daily steps were registered on 7 consecutive days every 4 weeks
Szmeja et al 2014 [66]	South Australia	Nested RCT	1108	Not specified	BMI ≥ 25	Between 10 and 20 weeks	Singleton	Not specified	Lifestyle advice group from (LIMIT) receive DVD or standard materials. Set goals. Received pregnancy book with nutrition + exercise in pregnancy book. *Comparison:* received the standard written materials and consultations	Self-report	Metabolic equivalent task units - MET (min/week)
^a^Vinter et al 2011 [55]	Denmark	RCT	304	Aged 18–40	BMI 30–45	Not specified	Not specified	Not specified	Two components: dietary counselling and PA. The aim was to limit GWG to 5 kg. Energy requirement was estimated and PA (30–60) min daily. Women also had free full time membership in a fitness centre. *Comparison:* received information about the content and purpose of the study with access to the website but no intervention	Objective	Aerobic Power Index submaximal aerobic exercise - VO2max
^a^Bruno et al 2017 [63]	Italy	Prospective RCT	191	Aged > 18	BMI ≥ 25	Not specified	Singleton	Not specified	PA intervention to develop a more active lifestyle (30mins of PA at least 3 times per week). *Comparison:* control group received a nutritional booklet which was in accordance with the Italian guidelines for diet and PA during pregnancy. All women the control group received antenatal care	Objective	Pedometer - Assess the number of steps and the duration of PA
^a^Van Horn et al 2018 [59]	USA	RCT	281	Aged 18–45	BMI 24–40	< 16 weeks	Singleton	Fluent in English, smartphone	Intervention prescribed calorie goals based on height, pre-conception weight, PA level and energy needs relevant for restricted total GWG. *Comparison:* usual care received access to MOMFIT website	Objective	Pedometer or smartphone tracking device and to log their activity, minutes of activity or steps per day
Kennelly et al 2018 [58]	Ireland	RCT	565	Aged 18–45	BMI > 25–39.9	Between 10 and 15 weeks	Not specified	Smartphone	Healthy lifestyle package, education session on nutrition and PA advice, healthy eating in pregnancy and benefits and safety of PA. Smartphone application reinforced the education and included 3 components; low glycaemic index recipes, exercise advice and nutritional exercise tips. *Comparison:* control group received standard antenatal care which in Ireland does not consist of any uniform advice	Self-report	International Physical Activity Questionnaire (IPAQ)

*RCT* randomised controlled trial, *MET* metabolic equivalent, *VO2* oxygen output, *PA* physical activity, *EE* energy expenditure, *D* dietary, *BMI* body mass index, *IPAQ* international physical activity questionnaire, *PPAQ* pregnancy physical activity questionnaire, *GDM* gestational diabetes mellitus, *T2D* type 2 diabetes, *GWG* gestational weight gain

^**a**^Significant reduction in maternal outcomes such as gestational weight gain and hypertension, and neonatal outcomes such as birth weight

### Intervention characteristics

Intervention duration ranged between 8 and 24 weeks. An explicit theoretical basis was mentioned in 6 out of the 19 studies, including stage theories of health decision making, behavioural modification, the trans-theoretical model, social cognitive theory and control theory [49, 51, 58, 60, 61, 66]. Most of the interventions were based in clinical settings [48, 49, 51, 52, 54, 55, 57–63, 65, 66], in the participants home [56, 64] or in a combination of both [50, 53]. Interventions were mostly delivered face-to-face and or via phone contact (phone calls, smartphone application) and were commonly provided by a physiotherapist, nutritionist/dieticians, study researchers, health educators or other health care professionals. The delivery of interventions ranged from at least one face-to face contact moment to phone contact throughout the intervention. One study did not specify contact type [56]. Table 2 provide details on the intervention components and BCTs in the included studies.

**Table 2:** Intervention characteristics

Author & Year	Theory	Contact type	Contact	Delivery	Setting	Type	Intervention duration^a^	BCTs
Callaway et al 2010 [48]	Not present	Face-to-face (individual) + via phone	6 face to face	Exercise physiologists; Dietician; Physiotherapists; Midwife	Clinical setting	PA	24 weeks	1.2 Problem solving 1.3 Goal setting outcome 2.2 Feedback on behaviour 2.3 Self-monitoring of behaviour 4.1 Instruction on how to perform behaviour 5.1 Information about health consequence
Oostdam et al 2012 [54]	Not present	Face-to-face (individual)	At least 1 face to face	Physiotherapist	Clinical setting + midwifery practices	PA	17 weeks (+ 12 weeks postpartum follow up)	3.1 Social Support (Unspecified) 4.1 Instruction on how to perform behaviour 5.1 Information about health consequence 8.1 Behavioural practice/rehearsal
Nascimento et al 2011 [53]	Not present	Face-to-face (individual + group)	8 face to face	Physicaltherapist	Clinical setting + participants home	PA	19 weeks	2.3 Self-monitoring of behaviour 3.1 Social Support (Unspecified) 4.1 Instruction on how to perform behaviour 5.1 Information about health consequence 8.1 Behavioural practice/rehearsal
Kong et al 2014 [50]	Not present	Face-to-face (individual)	3 face to face	Study coordinator	Clinical setting + participants home	PA	20 weeks	2.3 Self-monitoring of behaviour 4.1 Instruction on how to perform behaviour 12.5 Adding objects to the environment
Seneviratne et al 2016 [64]	Not present	Face-to-face (individual)	1 face to face	Exercise physiologist	Participants home	PA	15 weeks	1.1 Goal setting (behaviour) 4.1 Instruction on how to perform behaviour 12.5 Adding objects to the environment
Ong et al 2009 [56]	Not present	Not specified	no mention of contact with study team	Not specified	Participants home	PA	10 weeks	12.5 Adding objects to the environment
Santos et al 2005 [57]	Not present	Face-to-face (individual)	no mention of contact with study team	Not specified	Clinical setting	PA	12 weeks	8.1 Behavioural practice/rehearsal
Garnaes et al 2016 [65]	Not present	Face-to-face (individual or group)	At least 1 face to face	Physical therapist	Clinical setting	PA	19 weeks	2.3 Self-monitoring of behaviour 2.4 Self-monitoring of outcome(s) of behaviour 3.1 Social Support (Unspecified) 4.1 Instruction on how to perform behaviour 8.1 Behavioural practice/rehearsal
Dodd et al 2014 [60]	Stage theories of health decision making	Face-to-face (individual) + via phone	3 phone calls; 1 face to face	Dietician; Research assistants	Clinical setting	PA + diet	20 weeks (+ 16 weeks post-partum follow up)	1.2 Problem solving 1.3 Goal setting outcome 2.3 Self-monitoring of behaviour 4.1 Instruction on how to perform behaviour 5.1 Information about health consequence
Guelinckx et al 2009 [51]	Techniques of behavioural modification	Face-to-face (group)	3 group sessions	Nutritionist	Clinical setting	PA + diet	17 weeks	4.1 Instruction on how to perform behaviour 6.1 Demonstration of the behaviour
Hawkins et al 2015 [49]	The Trans theoretical Model and Social Cognitive Theory	Face-to-face (individual) + via phone	6 face to face; 5 phone calls	Health educators	Clinical setting	PA + diet	24 weeks (+ 6 weeks post-partum follow up)	1.2 Problem solving 1.3 Goal setting outcome 2.2 Feedback on behaviour 2.3 Self-monitoring of behaviour 3.1 Social Support (Unspecified)
Koivusalo et al 2016 [52]	Not present	Face-to-face (individual + group)	3 face to face; group visits	Study nurse; Nutritionist	Clinical setting	PA + diet	22 weeks	1.1 Goal setting (behaviour) 1.4 Action Planning 2.3 Self-monitoring of behaviour
Poston et al 2015 [61]	Control theory and elements of social cognitive theory	Face-to-face (individual + group)	8 face to face	Health trainer	Clinical setting	PA + diet	16 weeks (+ 24 week post-partum follow up)	1.2 Problem solving 1.3 Goal setting (outcome) 1.7 Review outcome goals 2.3 Self-monitoring of behaviour 3.1 Social Support (Unspecified) 4.1 Instruction on how to perform behaviour 5.1 Information about health consequence 6.1 Demonstration of the behaviour 6.2 Social comparison 8.1 Behavioural practice/rehearsal
Renault et al 2014 [62]	Not present	Face-to-face (individual) + via phone	6 face to face; 6 follow up calls	Dietician	Clinical setting	PA + diet	22 weeks	1.1 Goal setting (behaviour) 2.3 Self-monitoring of behaviour 3.1 Social Support (Unspecified)
Szmeja et al 2014 [66]	Stage theories of health decision making	Face-to-face (individual) + via phone	2 face to face; 3 calls	Research dietician; Trained research assistants	Clinical setting	PA + diet	8 weeks	1.2 Problem solving 1.3 Goal setting (outcome) 2.3 Self-monitoring of behaviour 4.1 Instruction on how to perform behaviour 5.1 Information about health consequence
Vinter et al 2011 [55]	Not present	Face-to-face (individual)	4 face to face	Dieticians; physiotherapists	Clinical setting	PA + diet	21 weeks	1.3 Goal setting (outcome) 2.3 Self-monitoring of behaviour 3.1 Social Support (Unspecified) 4.1 Instruction on how to perform behaviour 8.1 Behavioural practice/rehearsal
Bruno et al 2017 [63]	Not present	Face-to-face (individual)	At least 1 face to face	Gynaecologist; Dietician	Clinical setting	PA + diet	20 weeks	1.5 Review behaviour goal(s) 1.7 Review outcome goal(s) 2.3 Self-monitoring of behaviour 4.1 Instruction on how to perform behaviour
Van Horn et al 2018 [59]	Not present	Face-to-face (individual + group), email, text + phone	At least 1 face to face; 6 group sessions; weekly emails/phone call	Registered dietician nutritionist	Clinical setting, virtual setting (website)	PA + diet	20 weeks	1.2 Problem solving 2.2 Feedback on behaviour 2.3 Self-monitoring of behaviour 5.1 Information about health consequence
Kennelly et al 2018 [58]	Control theory and social cognitive theory	Face-to-face + smartphone application	3 face to face; email every 2 weeks	Nutritionist and obstetrician	Clinical setting, smartphone application	PA + diet	13 ± weeks	1.1 Goal setting (behaviour) 1.2 Goal setting (outcome) 2.4 Self-monitoring of outcomes(s) of behaviour 4.1 Instruction on how to perform behaviour 5.1 Information about health consequences 7.1 Prompts and cues 8.2 Behaviour substitution 8.3 Habit formulation

*PA* physical activity, *BCT* behaviour change technique, ^a^full intervention length

### Risk of bias assessment

**Fig. 2:**
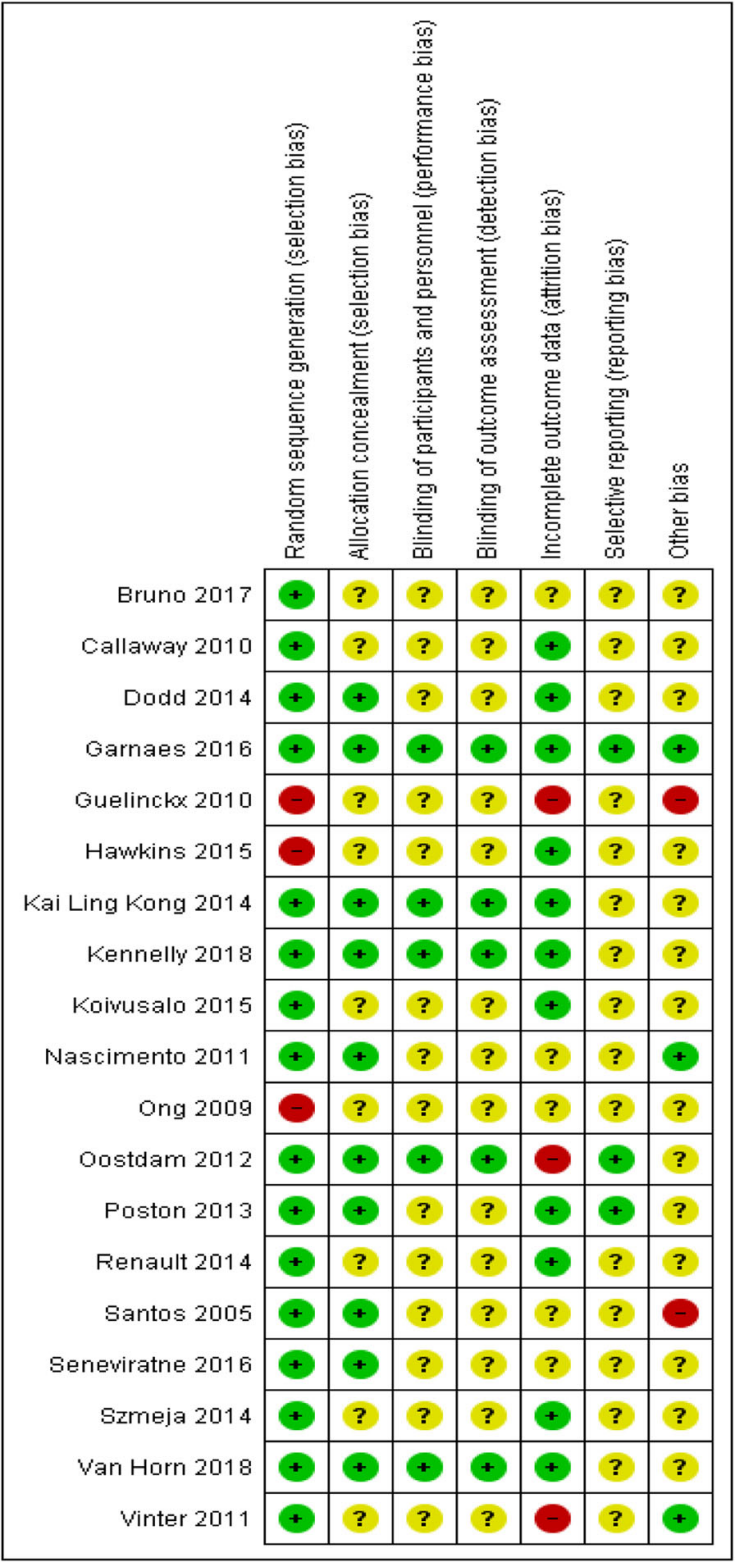
Risk of Bias

Overall risk of bias was high. Three studies were rated as having high potential risk of bias. Nine studies inadequately reported methodological quality indicators (e.g. studies lacked information on randomisation, allocation and outcome assessment concealment and inadequate missing data handling, see Additional file 2). For most studies, there was inadequate information to make judgements about methodological quality and the risk of bias. Seven studies were rated as low risk as they provided adequate information; however, five used self-report measures for physical activity. Furthermore, overall, blinding (performance bias and detection bias) was considered to have the highest risk as most studies failed to document the blinding procedures. A summary of the risk of bias for all 19 studies is shown in Fig. 2 (and Additional file 2). Studies were not excluded due to high risk and /or unclear risk of bias. Instead, sensitivity analyses were carried out for MET minutes per week and for step count data (see Additional file 3) in order to assess the influence of methodological quality on effect size. Sensitivity analysis was not conducted for VO_2_ max due insufficient data.

### Publication bias

For MET min per week, the Eggers test for bias was 2.51 [CI: 95% -3.08, 8.11] *p*-value = 0.314 which suggests that publication bias could not be detected. The funnel plot can be seen in Additional file 4. Eggers test and funnel plots were not conducted for step count data or VO_2_ max as insufficient data was available.

### Effectiveness of the intervention

#### Physical activity outcomes

**Fig. 3:**
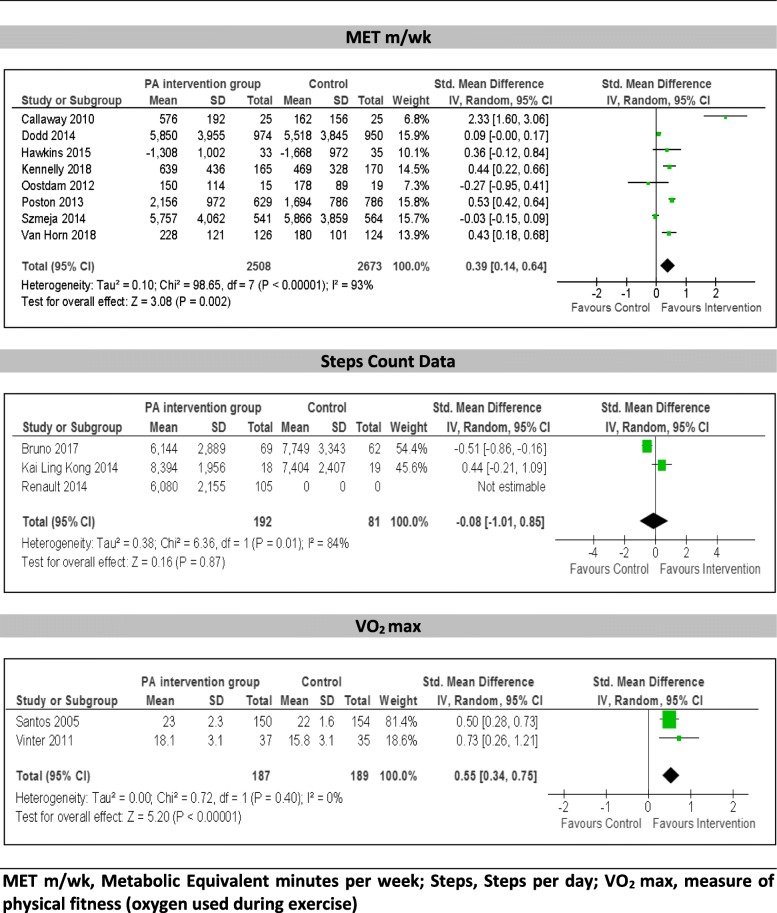
Meta-analysis of effect of interventions on physical activity outcomes

A wide variety of measures was used to assess physical activity in each of the included papers. Eight trials assessed physical activity objectively: four trials used pedometers deriving step-count [50, 59, 62, 63], one trial used an accelerometer to create metabolic equivalent (MET) [54], heart rate monitor data was collected to identify the duration and intensity of physical activity [64] and VO_2_ max was used as an indicator for physical fitness in two studies [55, 57]. Of the 19 included papers, 13 provided data suitable for inclusion in a meta-analysis [48–50, 54, 55, 57, 60–63, 66] (Fig. 3).

### Primary physical activity outcomes

#### Metabolic equivalent (MET) - minutes per week

Physical activity expressed in METS represents the metabolic equivalent intensity levels for activities with moderate intensity activity classified as 3–5 METS. Therefore 150 min of moderate intensity physical activity is equivalent to 450–750 MET/ minutes per week [67, 68]. Eight studies comparing interventions using METs minutes per week to a control group were combined in a meta-analysis [48, 49, 54, 58–61, 66]. A meta-analysis using standardised mean differences at follow up demonstrated a significant increase in MET minutes per week (SMD 0.39 [0.14, 0.64], Z = 3.08 *P* = 0.002). However, the studies were significantly heterogeneous (χ^2^ = 98.65, d.f. = 7 [*P* < 0.0001), I^2^ = 93%.

#### Step count data

Three studies comparing physical activity interventions to a control group that used step count data at follow up were combined (Fig. 3). One of these studies included multiple intervention arms which were combined, however participants in the control group of this study did not wear pedometers so step count data was not available for comparison [62]. The studies were significantly heterogeneous (χ^2^ = 6.36, d.f. = 1 [*P* = 0.01), I^2^ = 84% and demonstrated no significant difference in physical activity steps per day between the intervention and control groups at follow up (SMD -0.08 [− 1.01, 0.85], Z = 0.16 *P* = 0.87).

### Secondary physical activity outcome

#### VO_2_ max measures of physical fitness

Two studies compared VO_2_ max to measure the amount of oxygen used during exercise in order to assess physical fitness compared to control at follow up (Fig. 3). The studies were homogenous (χ^2^ = 0.72, d.f. = 1 [*P* = 0.40], I^2^ = 0%) and demonstrated significantly greater physical fitness in the intervention group compared to the control group (SMD 0.55 [0.34, 0.75], Z = 5.20 *P* = < 0.001).

#### Other physical activity interventions

Six additional trials that were not included in the meta-analyses due to insufficient data and different outcome measures reported varying intervention effects at follow up. Five of these studies reported an increase in physical activity or physical fitness for women in the intervention group compared to control [52, 53, 56, 64, 65]. Women who received diet and physical activity counselling increased their median weekly leisure time physical activity by 15 min (95% [C1 1–29 min] while the physical activity of women in the control group remained unchanged (*P* = 0.17 unadjusted) [52]. Furthermore, one home based intervention using a stationary bicycle, found that women in the intervention group improved their aerobic fitness by increasing the test time taken to reach target heart rate of 150 bpm (+ 48.0; *P* = 0.019) compared to the control group [64]. Similarly, another home based intervention found a trend towards increased fitness following the intervention (indicated by higher cycling power output 75% HR_max_) (*P* = 0.064, 57) compared to the control. A supervised exercise programme consisting of treadmill walking and resistance training found that the proportion of women reporting regular exercise training in late pregnancy was significantly higher in the exercise group than in the control group: 77 and 23% respectively (*P* < 0.01, 66). However, one study that consisted of two intervention groups (passive consisting of brochure and physical activity advice; active group consisting of the same but included active counselling) found physical activity significantly decreased from first trimester to the third trimester by 0.62 in the control group, by 0.33 in the active group and by 0.09 in the passive group (*p* = 0.002, 52).

#### Effect on health outcomes

Reductions in the incidence of GDM [52, 63], GWG [52, 55, 59] and the number of new-borns with a birth weight of > 4000 g was significantly lower in the intervention group [63] compared to controls.

### Behaviour change techniques

#### Presence of BCTs

A total of 19 different BCTs were applied within the 19 intervention studies, ranging between 1 and 10 in each study (Table 2). ‘Self-monitoring of behaviour’ and ‘Instruction on how to perform the behaviour’ were the most frequently described across the interventions and were identified in 13 out of the 19 studies (76.5%). Information about health consequences was used in 8 out of the 19 interventions (47.1%) and ‘social support (unspecified)’ was used in 7 out of the 17 interventions (41.2%), an average 11.1 times within each intervention (Table 3). ‘Social support (unspecified)’ and ‘Instruction on how to perform the behaviour’ were identified in one comparator group which consisted of once-weekly sessions of relaxation, respiratory exercises and light stretching and focus group discussions concerning maternity [57]. Inter-rater reliability was calculated by a chance-corrected kappa (k = 0.65) indicating substantial agreement.

**Table 3:** Frequencies of behaviour change techniques used in the interventions

Groups	BCT	Number	Percent	Average # of time BCT is used within each intervention^a^
Goals and planning	1.1 Goal setting (behaviour)	4	23.5	6
	1.2 Problem solving	6	35.3	8.5
	1.3 Goal setting outcome	7	41.2	4.3
	1.4 Action Planning	1	5.9	1
	1.5 Review behavioural goals	1	5.9	4
	1.7 Review outcome goals	1	5.9	5.5
Feedback and monitoring	2.2 Feedback on behaviour	3	17.6	12.3
	2.3 Self-monitoring of behaviour	13	76.5	5.5
	2.4 Self-monitoring of outcome of behaviour	2	11.8	1
Social support	3.1 Social Support (Unspecified)	7	41.2	11.1
Shaping Knowledge	4.1 Instruction on how to perform behaviour	13	76.5	9.1
Natural consequences	5.1 Information about health consequence	8	47.1	1.6
Comparison of behaviour	6.1 Demonstration of the behaviour	2	11.8	2
	6.2 Social comparison	1	5.9	8
Associations	7.1 Prompt and cues	1	5.9	1
Repetition and substitution	8.1 Behavioural practice/rehearsal	6	35.3	21
	8.2 Behaviour substitution	1	5.9	1
	8.3 Habit formation	1	5.9	1
Antecedents	12.5 Adding objects to the environment	3	17.6	1

*BCT* behaviour change technique

^a^estimated number of times a BCT was potentially implemented based on intervention description in each study and by calculating an average for each BCT

#### Number of BCTs and effect size

Subgroup analysis of which BCTs were associated with changes in physical activity outcome measures was not possible due to the small number of interventions included in the meta-analyses. The relationship between the total number of BCTs coded within an intervention and its effect size was found to be non-significant for MET (r = 0.20, *p* = 0.63) and for steps per day (r = 0.89, *p* = 0.31). Pearson’s r correlation coefficient was not calculated for VO_2_ max or for the other six studies not included in the meta-analyses due to insufficient data.

## Discussion

The aim of this review was to identify and summarise the evidence for the effectiveness of physical activity interventions for pregnant women with overweight and obesity on physical activity levels. Furthermore, it set out to identify which BCTs are used in these physical activity interventions. Following a systematic screening process, 19 physical activity intervention studies were included. Due to the variation of physical activity outcomes, 13 studies were included in the meta-analyses. Three small separate meta-analyses found a positive effect on MET minutes per week and VO_2_ max for improving physical activity during pregnancy. As described by Currie et al. 2013, physical activity tends to decrease gradually throughout pregnancy, therefore any outcome that demonstrates greater physical activity than control is deemed to be a desirable outcome [26]. Thus, the results of this review suggest that physical activity interventions are to some extent effective at increasing physical activity levels for women with overweight and obesity. However, these results should be viewed with caution as the pooled data came from studies that were highly heterogeneous. Despite physical activity reducing as pregnancy progresses due to the physical impediments experienced by women in the third trimester [69], some of the studies in this review established some positive physical activity results including an increase in physical fitness and a slight reduction in the incidence of GDM [52, 56, 64]. However, these results should also be approached as tentative due to small number of studies and a lack of available data.

Thirteen studies included in the three small separate meta-analyses found a main effect on physical activity outcomes for MET minutes per week and VO_2_ max but not for steps per day which suggests that some physical activity interventions could be a beneficial strategy for improving physical activity during pregnancy. Additionally, five other studies (not included in the meta-analysis) reported an increase in physical activity or physical fitness for women in the intervention group compared to control. As physical activity guidelines recommend participation in moderate intensity activity on ‘most days’ [8], this is a positive finding regarding the efficacy of these physical activity interventions. However, the low number of studies and the inclusion of three pilot trials suggest that caution should be applied when interpreting these results. The wide range of physical activity measures used within the interventions reviewed creates difficulty for researchers and health care professionals trying to draw conclusions. For interventions that include a self-report measure of physical activity, social desirability bias may have led to women over reporting their physical activity levels. Although the majority of self-report questionnaires were based on valid and reliable measures, objective measures such as accelerometers have demonstrated a higher degree of reproducibility and validity for quantifying duration and intensity of physical activity [70, 71].

In the current review, the most commonly used BCT categories within the interventions were ‘goals and planning’, ‘feedback and monitoring’, ‘social support’, ‘shaping knowledge’ and ‘natural consequences’. Other studies that have used the BCTs taxonomy to code lifestyle interventions in pregnancy have also found that categories such as ‘goals and planning’ and ‘feedback and monitoring’ were the most frequently used [31, 72, 73]. In this review, ‘self-monitoring of behaviour’ (using items such as diaries or workbooks to monitor physical activity) and ‘instruction on how to perform the behaviour’ (providing participants with descriptions for particular exercises) emerged as the most frequently used BCTs across the interventions. Interventions which included these BCTs showed some positive effects but further research is required to examine the link between BCTs and intervention effectiveness. Research involving adults with overweight and obesity, also identified ‘self-monitoring of behaviour’ as a common BCT in physical activity interventions [74]. Furthermore, a review examining the use of pedometers to increase physical activity, found significant increases in physical activity in an adult population [75]. In pregnancy, women with overweight and obesity have indicated that pedometers and step counts could help with self-monitoring [76] with pedometers being found as an acceptable form of self-monitoring [77]. Therefore, based on the results from this review and previous research, future interventions should include some component of self-monitoring in order to improve physical activity levels for pregnant women with overweight and obesity. While the BCTs used to promote physical activity in this review correspond closely to those found in previous antenatal interventions [31, 72], the identification of ‘social support’ is new to this pregnant population with overweight and obesity, with other systematic reviews of antenatal interventions failing to identify this BCT. Previous research has identified ‘social influences’ as an enabler to physical activity for women with overweight and obesity [76]. Furthermore, another study which investigated women’s experiences of pregnancy found that physically active women faced some criticism from family members about their active lifestyles [78]. Thus, future interventions need to take into account the woman’s social support network, to include family, friends and other pregnant women in these antenatal interventions. As previously found, this result highlights the importance of selecting appropriate BCTs for each population and not assuming all BCTs will be equally effective.

### Strengths and limitations

This systematic review was comprehensive in its scope and search and was conducted in accordance with the PRISMA (preferred reporting items for systematic reviews and meta-analysis) statement [41]. A strength of this study was the use of an established instrument (BCTTv1) to systematically code the presence of BCTs in physical activity interventions for pregnant women with overweight and obesity.

The main limitations of this review stem from the inadequate reporting of physical activity data and poor intervention designs. Large differences in the type of activity measured, along with self-report measures highlights a limitation of the literature to date, making comparisons challenging. Also the use of physical fitness as a secondary outcome can be difficult to interpret. The studies lacked sufficient data to calculate pooled effect sizes for all physical activity outcome measures. Furthermore, while publication bias was not detected or performed for all outcomes, the majority of studies were of high risk of bias. Due to the small number of studies included in the meta-analysis and the high degree of heterogeneity, caution must be applied when generalising these findings. Therefore, the evidence base is weak and calls for more robust studies. Future research using robust high quality studies will foster better data to inform policy and practice.

The majority of interventions were based in a clinical setting which may have impacted intervention effectiveness. Furthermore, physical activity data were assessed using the last measure before birth (between 28 and 35 weeks’ gestation) thus reducing comparability between studies with follow up ranging from 8 weeks’ gestation to 12 months postpartum. Also, there were differences in the delivery modes and person, the intensity of the interventions and how active the women were prior to the intervention which may have also played a role in intervention effectiveness (and the BCTs used). As pregnancy progresses women tend to become less active [79], thus, future research is required to assess trimester (stage of pregnancy) and whether this impacts intervention effectiveness and the BCT employed.

Results from this review can be considered exploratory as no conclusions regarding the potential relationship between intervention content and effectiveness can be made. This was due to the paucity of intervention studies. A higher number of RCT studies of physical activity interventions for women with overweight and obesity during pregnancy are needed to draw firm conclusions. Many studies failed to provide adequate information on intervention content. As described by others, studies do not always provide adequate intervention content [80]. Not all studies had associated methods or protocol papers available making it possible that other BCTs were used but not coded. This, however, is a common problem conducting reviews such as these [28, 34, 81]. Furthermore, correlation of BCTs and outcomes has previously been identified as a methodological weakness [82]. It is difficult to know if routine antenatal care provided a BCT or not. In order to reliably identify the BCTs associated with physical activity for women with overweight and obesity, control groups identified as routine care should be described in intervention reports and coded for BCTs. Furthermore, as one control group contained BCTs, this creates a potential source of bias affecting the reliability of the data. Fidelity was poorly reported so it was impossible to determine if BCTs were delivered or received as intended.

Some of the BCT definitions were difficult to interpret, in particular ‘Information about health consequence’. This definition was not explicit about whether ‘health consequences’ related to the positive or negative health outcomes of performing or not preforming the behaviour, respectively. Therefore, after detailed discussion ‘Information about health consequence’ was coded for both. Furthermore, intervention components such as free gym membership and swimming pool vouchers were used within two intervention studies [52, 55] and were not coded as BCTs; however these components could have an impact on behaviour change. In addition, contextual factors shape interventions and, therefore can influence how BCTs are delivered. Context can include individuals, teams, organisational structures and cultures, resources, leadership styles and relationships [83, 84].

Future interventions need to clearly define and report the behavioural outcome measure for physical activity such as core outcome sets for physical activity in pregnancy [85, 86]. Furthermore, future intervention should follow TIdieR guidelines for reporting intervention content [87]. Moreover, interventions need to provide more transparent and comprehensive descriptions of BCTs used, and should include detail of context, fidelity, dose and clarity regarding the theory used within the intervention. Improved intervention description including the use of recognised and standardised taxonomies would increase ability to assess the BCTs and to examine the relationship between technique usage and change in physical activity. Despite these limitations, it is important to conduct such reviews enabling researchers to describe and analyse in detail the content of interventions, aiding the accuracy and communication required to build a cumulative evidence base [88].

## Conclusion

The meta-analysis and narrative description of the included studies in this review revealed an increase in physical activity or physical fitness for pregnant women with overweight and obesity. A range of BCTs that could be used to help improve physical activity levels during pregnancy were identified, including: ‘goals and planning’, ‘feedback and monitoring’ and ‘shaping knowledge’ with ‘social support’ being newly identified for this population. Given the importance of physical activity to many subsequent outcomes in pregnancy, an explicit theoretical basis is needed for intervention development. Furthermore, interventions need to not only report the presence and frequency of BCTs but also the intensity and quality in which they are delivered or implemented. As ‘social support’ was identified within this review for a pregnant population with overweight or obesity future interventions need to take into account woman’s social support networks, to include family and friends. These conclusions are tentative because of the high risk of bias of the included studies. Therefore, future studies should consider physical activity outcome carefully so that studies can be meaningfully compared. Intervention developers need to use recognised and standardised taxonomies to describe intervention content. To enable us to identify which BCTs are most effective for physical activity interventions with pregnant women with who are overweight and obese.

## Supplementary information

**Additional file 1: Table S1.** Database searches.

**Additional file 2: Table S2.** Methodological quality rating.

**Additional file 3: Figure S1.** Sensitivity Analysis.

**Additional file 4: Figure S2.** Funnel plot.

## Data Availability

All data generated during this study are included in this published article [and its supplementary information files].

## Abbreviations

BCT: Behaviour change technique
GDM: Gestational diabetes mellitus
MET: Metabolic equivalent
PA: Physical activity
RCT: Randomised controlled trial
VO_2_ max: Maximal oxygen uptake
