# Supplementary material for: Effectiveness of physical activity interventions for overweight and obesity during pregnancy: a systematic review of the content of behaviour change interventions
Source: Int J Behav Nutr Phys Act. 2019 Nov 1;16:97. doi: 10.1186/s12966-019-0859-5 (PMC6825353; doi:10.1186/s12966-019-0859-5)
Supplement: Supplementary file 1 — Additional file 1: Table S1. Database searches. [file 12966_2019_859_MOESM1_ESM.docx]

**Additional file 1**

**Table S1:** Searches ***MEDLINE, PsycINFO, SPORTDiscuss, CINAHL, PEDro, Cochrane Library, EMBASE and PubMed*** from database inception to Jan 2018

**Table 1: Medline**

| **Search terms** | **No of records returned** | **Updated search** | **Updated**  **Search** |
| --- | --- | --- | --- |
| **For *MEDLINE* *(1879 – Jan 2018)*** | ***June 2016*** | ***Jan 2018*** | ***Jan 2019*** |
| 1. (MH “Pregnancy”) | 7769819 |  |  |
| 1. (MH “Pregnant Women”) | 5638 |  |  |
| 1. (MH “Prenatal Care”) | 21932 |  |  |
| 1. **[#1 or #2 or #3]** | **772027** | **18994** | **11854** |
| 1. (MH “Diabetes, Gestational”) | 6955 |  |  |
| 1. (MH “Body Weight”) | 169788 |  |  |
| 1. (MH “Weight Gain”) | 25146 |  |  |
| 1. (MH “Overweight”) | 15555 |  |  |
| 1. (MH “Obesity”) | 140600 |  |  |
| 1. (MH “Body Mass Index”) | 92887 |  |  |
| 1. **[#5 or #6 or #7 or #8 or #9 or #10]** | **372507** | **52107** | **8550** |
| 1. **[#4 and #11]** | **31215** | **1687** | **620** |
| 1. “behav*” | 1347420 |  |  |
| 1. “behavio#r” | 1042408 |  |  |
| 1. (MH “Health Behavior”) | 38336 |  |  |
| 1. (MH “Life Style”) | 46886 |  |  |
| 1. “intervention” | 398591 |  |  |
| 1. “program#e” | 90086 |  |  |
| 1. “change” | 854663 |  |  |
| 1. **[#13 or #14 or #15 or #16 or #17 or #18 or #19]** | **2507332** | **282446** | **313180** |
| 1. **[#12 and #20]** | **5634** | **426** | **236** |
| 1. (MH “Motor Activity”) | 85382 |  |  |
| 1. (MH “Exercise”) | 76163 |  |  |
| 1. (MH “Physical Exertion”) | 53932 |  |  |
| 1. (MH “Walking”) | 23587 |  |  |
| 1. (MH “Movement”) | 63119 |  |  |
| 1. (MH “Exercise Therapy”) | 29951 |  |  |
| 1. (MH “Leisure Activities”) | 7039 |  |  |
| 1. (MH “Physical Fitness”) | 23486 |  |  |
| 1. “physical activity” | 74394 |  |  |
| 1. **[#22 or #23 or #24 or #25 or #26 or #27 or #28 or #29 or #30]** | **362699** | **402050** | **18550** |
| 1. **[#21 and #31]** | **1031** | **88** | **37** |
| MH –Exact subject heading  “…” – free text term (used when no exact subject heading was available)  *truncation  #wildcard | |  |  |

**Table 2: PsycINFO**

| **Search terms** | **No of records returned** | **Updated search** | **Updated search** |
| --- | --- | --- | --- |
| **For *PsycINFO (1967 – Jan 2018)*** | ***June 2016*** | ***Jan 2018*** | ***Jan 2019*** |
| 1. Pregnancy | 35376 |  |  |
| 1. Pregnant Women | 8301 |  |  |
| 1. Prenatal Care | 2735 |  |  |
| 1. **[#1 or #2 or #3]** | **37814** | **3442** | **2086** |
| 1. Diabetes, Gestational | 367 |  |  |
| 1. Body Weight | 22718 |  |  |
| 1. Weight Gain | 9237 |  |  |
| 1. Overweight | 11502 |  |  |
| 1. Obesity | 30577 |  |  |
| 1. Body Mass Index | 15018 |  |  |
| 1. **[#5 or #6 or #7 or #8 or #9 or #10]** | **59759** | **6036** | **3866** |
| 1. **[#4 and #11]** | **2143** | **306** | **204** |
| 1. behav* | 1287789 |  |  |
| 1. behavio#r | 1001457 |  |  |
| 1. Health Behavior | 51063 |  |  |
| 1. Life Style | 25001 |  |  |
| 1. Intervention | 312629 |  |  |
| 1. Program#e | 21480 |  |  |
| 1. Change | 492798 |  |  |
| 1. **[#13 or #14 or #15 or #16 or #17 or #18 or #19]** | **1763830** | **132323** | **85545** |
| 1. **[#12 and #20]** | **1263** | **184** | **124** |
| 1. Motor Activity | 10364 |  |  |
| 1. Exercise | 62196 |  |  |
| 1. Physical Exertion | 407 |  |  |
| 1. Walking | 12640 |  |  |
| 1. Movement | 133178 |  |  |
| 1. Exercise Therapy | 1390 |  |  |
| 1. Leisure Activities | 5400 |  |  |
| 1. Physical Fitness | 5242 |  |  |
| 1. physical activity | 30421 |  |  |
| 1. **[#22 or #23 or #24 or #25 or #26 or #27 or #28 or #29 or #30]** | **230044** | **18938** | **11341** |
| 1. **[#21 and #31]** | **228** | **43** | **28** |
| Free text terms searched  *truncation  #wildcard | |  |  |

**Table 3: SPORTDiscus**

| **Search terms** | **No of records returned** | **Updated search** | **Updated search** |
| --- | --- | --- | --- |
| **For *SPORTDiscus (1830 – Jan 2018)*** | ***June 2016*** | ***Jan 2018*** | ***Jan 2019*** |
| 1. Pregnancy | 8241 |  |  |
| 1. Pregnant Women | 2916 |  |  |
| 1. Prenatal Care | 450 |  |  |
| 1. **[#1 or #2 or #3]** | **9005** | **510** | **386** |
| 1. Diabetes, Gestational | 228 |  |  |
| 1. Body Weight | 18175 |  |  |
| 1. Weight Gain | 3401 |  |  |
| 1. Overweight | 7189 |  |  |
| 1. Obesity | 20105 |  |  |
| 1. Body Mass Index | 12323 |  |  |
| 1. **[#5 or #6 or #7 or #8 or #9 or #10]** | **44285** | **4698** | **3204** |
| 1. **[#4 and #11]** | **1014** | **105** | **92** |
| 1. behav* | 81237 |  |  |
| 1. behavio#r | 62291 |  |  |
| 1. Health Behavior | 10222 |  |  |
| 1. Life Style | 16086 |  |  |
| 1. Intervention | 40752 |  |  |
| 1. Program#e | 13772 |  |  |
| 1. Change | 103024 |  |  |
| 1. **[#13 or #14 or #15 or #16 or #17 or #18 or #19]** | **218733** | **19173** | **13495** |
| 1. **[#12 and #20]** | **342** | **41** | **44** |
| 1. Motor Activity | 2400 |  |  |
| 1. Exercise | 208796 |  |  |
| 1. Physical Exertion | 604 |  |  |
| 1. Walking | 19190 |  |  |
| 1. Movement | 55312 |  |  |
| 1. Exercise Therapy | 6264 |  |  |
| 1. Leisure Activities | 4566 |  |  |
| 1. Physical Fitness | 97156 |  |  |
| 1. physical activity | 49096 |  |  |
| 1. **[#22 or #23 or #24 or #25 or #26 or #27 or #28 or #29 or #30]** | **328358** | **21461** | **17115** |
| 1. **[#21 and #31]** | **135** | **22** | **25** |
| Free-text terms  *truncation  #wildcard | |  |  |

**Table 4: CINAHL**

| **Search terms** | **No of records returned** | **Updated search** | **Updated search** |
| --- | --- | --- | --- |
| **For *CINAHL (1961 – Jan 2018)*** | ***June 2016*** | ***Jan 2018*** | ***Jan 2019*** |
| 1. (MH “Pregnancy”) | 132956 |  |  |
| 1. Pregnant Women | 16790 |  |  |
| 1. (MH “Prenatal Care”) | 11024 |  |  |
| 1. **[#1 or #2 or #3]** | **138574** | **12262** | **9453** |
| 1. Diabetes, Gestational | 4922 |  |  |
| 1. (MH “Body Weight”) | 19893 |  |  |
| 1. (MH “Weight Gain”) | 8117 |  |  |
| 1. Overweight | 15053 |  |  |
| 1. (MH “Obesity”) | 54070 |  |  |
| 1. (MH “Body Mass Index”) | 54938 |  |  |
| 1. **[#5 or #6 or #7 or #8 or #9 or #10]** | **116422** | **12847** | **9709** |
| 1. **[#4 and #11]** | **9422** | **1294** | **976** |
| 1. behave* | 251247 |  |  |
| 1. behavio#r | 195628 |  |  |
| 1. (MH “Health Behavior”) | 32472 |  |  |
| 1. (MH “Life Style”) | 18243 |  |  |
| 1. intervention | 227087 |  |  |
| 1. program#e | 25825 |  |  |
| 1. change | 264424 |  |  |
| 1. **[#13 or #14 or #15 or #16 or #17 or #18 or #19]** | **663024** | **116151** | **90695** |
| 1. **[#12 and #20]** | **2065** | **394** | **339** |
| 1. (MH “Motor Activity”) | 8945 |  |  |
| 1. (MH “Exercise”) | 32620 |  |  |
| 1. Physical Exertion | 289 |  |  |
| 1. (MH “Walking”) | 14898 |  |  |
| 1. (MH “Movement”) | 9748 |  |  |
| 1. Exercise Therapy | 2954 |  |  |
| 1. (MH “Leisure Activities”) | 5444 |  |  |
| 1. (MH “Physical Fitness”) | 11512 |  |  |
| 1. (MH“physical activity”) | 24083 |  |  |
| 1. **[#22 or #23 or #24 or #25 or #26 or #27 or #28 or #29 or #30]** | **97804** | **10113** | **9198** |
| 1. **[#21 and #31]** | **337** | **58** | **40** |
| MH –Exact subject heading  “…” – free text term (used when no exact subject heading was available)  *truncation #wildcard | |  |  |

**Table 5: PEDro**

| **Search terms** | **No of records returned** | **Updated search** | **Updated search** |
| --- | --- | --- | --- |
| **For *PEDro (1999 – Jan 2018)*** | ***June 2016*** | ***Jan 2018*** | ***Jan 2019*** |
| 1. Pregnan* |  |  |  |
| 1. Clinical trial |  |  |  |
| 1. **[#1 and #2]** | **399** | **56** | **37** |
| (Database advanced search difficult to work with so kept search strategy simple to capture everything on pregnancy and trials) *truncation | |  |  |

**Table 6: Cochrane**

| **Search terms** | **No of records returned** | **Updated search** | **Updated search** |
| --- | --- | --- | --- |
| **For *Cochrane library (1993 – Jan 2018)*** | ***June 2016*** | ***Jan 2018*** | ***Jan 2019*** |
| 1. MeSH descriptor: [Pregnancy] | 6321 |  |  |
| 1. MeSH descriptor: [Pregnant Women] | 122 |  |  |
| 1. MeSH descriptor: [Prenatal Care] | 1203 |  |  |
| 1. **#1 or #2 or #3** | **7210** | **678** | **450** |
| 1. MeSH descriptor: [Diabetes, Gestational] | 459 |  |  |
| 1. MeSH descriptor: [Body Weight] | 18942 |  |  |
| 1. MeSH descriptor: [Weight Gain] | 1876 |  |  |
| 1. MeSH descriptor: [Overweight] | 10086 |  |  |
| 1. MeSH descriptor: [Obesity] | 9187 |  |  |
| 1. MeSH descriptor: [Body Mass Index] | 7517 |  |  |
| 1. **#5 or #6 or #7 or #8 or #9 or #10** | **22600** | **917** | **4320** |
| 1. **#4 and #11** | **707** | **481** | **63** |
| 1. MeSH descriptor: [Health Behavior] | 17663 |  |  |
| 1. MeSH descriptor: [Life Style] | 3530 |  |  |
| 1. intervention | 115332 |  |  |
| 1. behav* | 77578 |  |  |
| 1. behavio?r | 11697 |  |  |
| 1. program?e | 16593 |  |  |
| 1. change 9 | 90017 |  |  |
| 1. **#13 or #14 or #15 or #16 or #17 or #18 or #19** | **235528** | **1675** | **58072** |
| 1. **#12 and #20** | **289** | **480** | **37** |
| 1. MeSH descriptor: [Motor Activity] | 19579 |  |  |
| 1. MeSH descriptor: [Exercise] | 16790 |  |  |
| 1. MeSH descriptor: [Physical Exertion] | 3505 |  |  |
| 1. MeSH descriptor: [Walking] | 3151 |  |  |
| 1. MeSH descriptor: [Movement] | 23338 |  |  |
| 1. MeSH descriptor: [Exercise Therapy] | 9086 |  |  |
| 1. MeSH descriptor: [Leisure Activities] | 13852 |  |  |
| 1. MeSH descriptor: [Physical Fitness] | 2444 |  |  |
| 1. MeSH descriptor: [Motor Activity] | 19579 |  |  |
| 1. **#22 or #23 or #24 or #25 or #26 or #27 or #28 or #29 or #30** | **35152** | **739** | **2920** |
| 1. **#21 and #31** | **60** | **254** | **11** |
| MeSH descriptor (Medical subject heading)  *truncation  ?wildcard | |  |  |

**Table 7: EMBASE**

| **Search terms** | **No of records returned** | **Updated search** | **Updated search** |
| --- | --- | --- | --- |
| **For *EMBASE (1947 – Jan 2018)*** | ***June 2016*** | ***Jan 2018*** | ***Jan 2019*** |
| 1. ‘Pregnancy’/exp | 654342 |  |  |
| 1. ‘Pregnant Women’/exp | 46339 |  |  |
| 1. ‘Prenatal Care’/exp | 121687 |  |  |
| 1. **[#1 or #2 or #3]** | **732085** | **70953** | **33740** |
| 1. ‘Diabetes, Gestational’/exp | 24837 |  |  |
| 1. ‘Body Weight’/exp | 492033 |  |  |
| 1. ‘Weight Gain’/exp | 77604 |  |  |
| Overweight | / |  |  |
| 1. ‘Obesity’/exp | 366253 |  |  |
| 1. ‘Body Mass’/exp | 245340 |  |  |
| 1. **[#5 or #6 or #7 or #8 or #9]** | **900504** | **122083** | **103968** |
| 1. **[#4 and #11]** | **78264** | **9158** | **7285** |
| 1. behav* | 1739263 |  |  |
| 1. behavio?r | 219723 |  |  |
| 1. ‘Health Behavior’/exp | 304440 |  |  |
| 1. ‘Life Style’/exp | 85097 |  |  |
| 1. Intervention | 629575 |  |  |
| 1. Program?e | 128868 |  |  |
| 1. Change | 1075019 |  |  |
| 1. **[#12 or #13 or #14 or #15 or #16 or #17 or #18]** | **3447254** | **361533** | **290840** |
| 1. **[#11 and #19]** | **13558** | **1943** | **1603** |
| 1. ‘Motor Activity’/exp | 424415 |  |  |
| 1. ‘Exercise’/exp | 253718 |  |  |
| Physical Exertion | / |  |  |
| 1. ‘Walking’/exp | 79603 |  |  |
| 1. ‘Movement (physiology)’ /exp | 317771 |  |  |
| 1. ‘Kinesiotherapy’/exp | 57036 |  |  |
| 1. ‘Leisure’/exp | 24099 |  |  |
| 1. ‘Fitness’ /exp | 33137 |  |  |
| 1. ‘physical activity’ /exp | 286628 |  |  |
| 1. **[#22 or #23 or #24 or #25 or #26 or #27 or #28 or #29 or #30]** | **1087669** | **98229** | **89584** |
| 1. **[#21 and #31]** | **1919** | **476** | **276** |
| Not available as Emtree terms – Overweight/ Physical Exertion   - ‘Movement (physiology)’ - movement - ‘Kinesiotherapy’ – exercise therapy   *truncation  ?wildcard | |  | **Articles 153 (Embase only 45)** |

**Table 8: PubMed**

| **Search terms** | **No of records returned** | **Updated search** | **Updated search** |
| --- | --- | --- | --- |
| **For *PubMed (1996 – Jan 2018)*** | ***June 2016*** | ***Jan 2018*** | ***Jan 2019*** |
| 1. Pregnancy | 848096 |  |  |
| 1. Pregnant Women | 87616 |  |  |
| 1. Prenatal Care | 36250 |  |  |
| 1. **[#1 or #2 or #3]** | **861750** | **20370** | **30445** |
| 1. Diabetes, Gestational | 15138 |  |  |
| 1. Body Weight | 525646 |  |  |
| 1. Weight Gain | 68353 |  |  |
| 1. Overweight | 181872 |  |  |
| 1. Obesity | 240033 |  |  |
| 1. Body Mass Index | 172157 |  |  |
| 1. **[#5 or #6 or #7 or #8 or #9 or #10]** | **698688** | **20763** | **54353** |
| 1. **[#4 and #11]** | **72005** | **2425** | **4439** |
| 1. behav* | 1424816 |  |  |
| 1. behaviour | 1845255 |  |  |
| 1. Health Behavior | 330291 |  |  |
| 1. Lifestyle | 121976 |  |  |
| 1. Intervention | 427292 |  |  |
| 1. Programme | 89461 |  |  |
| 1. Change | 857962 |  |  |
| 1. **[#13 or #14 or #15 or #16 or #17 or #18 or #19]** | **3289519** | **809804** | **643030** |
| 1. **[#12 and #20]** | **16973** | **1691** | **2994** |
| 1. Motor Activity | 267074 |  |  |
| 1. Exercise | 319266 |  |  |
| 1. Physical Exertion | 56892 |  |  |
| 1. Walking | 59762 |  |  |
| 1. Movement | 583411 |  |  |
| 1. Exercise Therapy | 88149 |  |  |
| 1. Leisure Activities | 188085 |  |  |
| 1. Physical Fitness | 33970 |  |  |
| 1. physical activity | 359556 |  |  |
| 1. **[#22 or #23 or #24 or #25 or #26 or #27 or #28 or #29 or #30]** | **985231** | **90904** | **70725** |
| 1. **[#21 and #31]** | **2733** | **185** | **326** |
| MeSH terms limited the result numbers so free txt terms where searched  #? Wildcard not available in PubMed | |  | **Full text 142** |
