# Supplementary material for: Effectiveness of physical activity interventions for overweight and obesity during pregnancy: a systematic review of the content of behaviour change interventions
Source: Int J Behav Nutr Phys Act. 2019 Nov 1;16:97. doi: 10.1186/s12966-019-0859-5 (PMC6825353; doi:10.1186/s12966-019-0859-5)
Supplement: Supplementary file 2 — Additional file 2: Table S2. Methodological quality rating. [file 12966_2019_859_MOESM2_ESM.docx]

**Table S2: Risk of Bias Assessment**

| **Reference** | **Sequence generation** | **Concealed allocation** | **Outcome assessment** | **Retention rate** | **Missing data handling** | **Other bias** | **Overall risk of bias** | **Rational/ notes** |
| --- | --- | --- | --- | --- | --- | --- | --- | --- |
| **Callaway et al 2015** | A | U | U | 70% | U | U | Unclear | Random number allocation technique conducted by third party at another location; Unclear information across most areas |
| **Dodd et al 2014** | A | A | U | 86% | A | U | Low | Telephone central randomisation service which utilises a computer generated schedule with balanced variable blocks; Intention to treat basis; Women were aware of the treatment allocation; Generally well conducted RCT with PA measures; Recall bias |
| **Hawkins et al 2015** | N | U | U | 77% | A | U | Unclear | Randomised by the health educators; Intention to treat  Unclear information across most areas; Small sample size; Recall bias |
| **Kong et al 2014** | A | A | A | 88* | U | U | Low | Assigned to intervention/control using a computer based random sample generator; No detail on missing data; Generally well documented; small sample size and high variance |
| **Oostdam et al 2012** | A | A | A | 40% | A | U | High | Computerised random number generator; After baseline measurement – women were informed about their group allocation; Research and research staff will not be blinded but all independent examiners will be unaware of group allocation; Intention to treat; High dropout rate |

| **Reference** | **Sequence generation** | **Concealed allocation** | **Outcome assessment** | **Retention rate** | **Missing data handling** | **Other bias** | **Overall risk of bias** | **Rational/ notes** |
| --- | --- | --- | --- | --- | --- | --- | --- | --- |
| **Poston et al 2015** | A | A | U | 90% | A | U | Low | Computer generated randomisation procedure; Intention to treat; Missing at random assumption; Self-report |
| **Renualt et al 2014** | A | U | U | 91% | U | U | Unclear | Randomised by a dietician – web allocation by an independent organisation; Intention to treat approach; Unclear information across most areas; no data from control group after the intervention; homogeneity – lowers external validity |
| **Szmeja et al 2014** | A | U | U | 99% | U | U | Unclear | Telephone central randomisation service – computer generated schedule; Intention to treat; Unclear information across most areas |
| **Vinter et al 2011** | A | U | U | 20% | U | A | High | Randomisation computer generated numbers in closed envelopes; Selection Bias |
| **Santos et al 2005** | A | A | U | 78% | U | N | High | Randomised using a block sequence generator from a random number table by a statistician; Un-blinded program of supervised PA; Intention to treat; Attrition during the follow up was relatively high |
| **Ong et al 2009** | N | U | U | U | U | U | Unclear | Participants were randomly allocated – no detail; Unclear information across most areas |
| **Guelinckx et al 2009** | N | U | U | 62% | U | N | Unclear | Randomly allocated – no detail; Recall bias; Unclear information across most areas |

| **Reference** | **Sequence generation** | **Concealed allocation** | **Outcome assessment** | **Retention rate** | **Missing data handling** | **Other bias** | **Overall risk of bias** | **Rational/ notes** |
| --- | --- | --- | --- | --- | --- | --- | --- | --- |
| **Koivusalo et al 2016** | A | U | U | 91% | U | U | Unclear | Randomly permuted process – the randomisation process was performed by a study nurse and by dispensing the next sequentially numbered subject code and opening the corresponding code envelope; Unclear information across most areas |
| **Garnaes et al 2016** | A | A | A | 81% | A | A | Low | Randomly allocated using a computer random number generator; Statistician was blinded; Weight measurement and blood’s completed by blinded personnel; All other assessment were done non-blinded; Intention to treat; per protocol analysis – only included the women in the exercise group who adhered to the exercise protocol; selection bias |
| **Nascimento et al 2011** | A | A | U | 97% | U | A | Low | Randomised using statistical programme which generated a list of random numbers based on a uniform distribution; To ensure blinding, the sequence was randomly distributed in opaque envelopes; Analysis by treatment schedule (intention to treat); Self-report bias |
| **Seneviratne et al 2016** | A | A | U | N/A | U | U | Unclear | Two arm randomisation 1;1 allocation ratio – randomization sequence generated by biostatistician not related to the study and were used sequentially according to enrolment order; Group allocation was revealed to participants after baseline assessments recruitment coordinator did not have access to the allocations; Intention to treat; Unclear information across most areas |
| **Reference** | **Sequence generation** | **Concealed allocation** | **Outcome assessment** | **Retention rate** | **Missing data handling** | **Other bias** | **Overall risk of bias** | **Rational/ notes** |
| **Bruno et al 2017** | A | U | U | 68% | U | U | Unclear | Computer generated random allocation sequence; Not blinded to group allocation |
| **Van Horn et al 2018** | A | A | A | 98% | A | U | Low | Women were randomized at a 1:1 allocation in random blocks of four and six by 16 weeks gestation, randomisation was sequentially blinded with statistician provided allocation stored in a Microsoft access database, once eligibility was confirmed the study coordinator randomised participants and forwarded intervention group status to the registered dietician nutritionist. Blinded study personnel collected all data at baseline, 24, 35 weeks. Trained and certified staff was blinded to treatment assignment and prior data collections to minimize potential bias; intention to treat principle; no adjusted for an inflated type 1 error; some reporting bias in follow up diet data. |
| **Kennelly et al 2018** | A | A | A | 88% | U | U | Low | Randomization was performed using a computer- generated sequence in a ratio of one to one. The biostatistician prepared sequentially numbered, sealed opaque envelopes, which were opened at the first study visit. As a result of the nature of the intervention, neither participants nor researchers were blinded to the intervention or outcomes. Intention to treat. |

A: Adequate, N: Not Adequate, U: Unclear, N/A: Not applicable
