# Supplementary material for: Effectiveness of physical activity interventions for overweight and obesity during pregnancy: a systematic review of the content of behaviour change interventions
Source: Int J Behav Nutr Phys Act. 2019 Nov 1;16:97. doi: 10.1186/s12966-019-0859-5 (PMC6825353; doi:10.1186/s12966-019-0859-5)
Supplement: Supplementary file 3 — Additional file 3: Figure S1. Sensitivity Analysis. [file 12966_2019_859_MOESM3_ESM.docx]

**Figure S1: Sensitivity analysis**

**MET m/wk**

**Low risk studies only^1^**


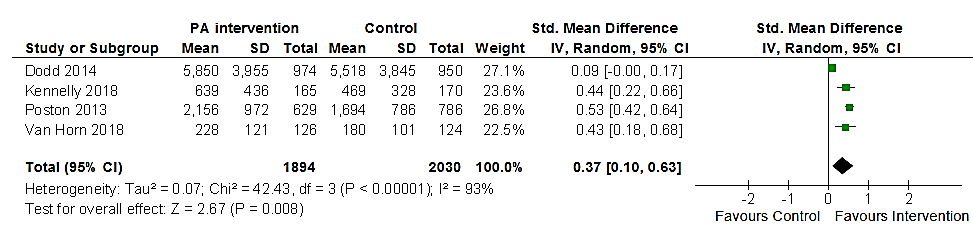


^1^ Removing high risk Oostdam et al (2012) and unclear risk Callaway et al (2010), Hawkins et al (2015) and Szmeja et al (2014)

A meta-analysis for MET minutes per week including low risk studies only, demonstrated a significant increase in MET minutes per week (SMD 0.37 [0.10, 0.63], Z = 2.67 P = 0.008). However, the studies were significantly heterogeneous (χ^2^ = 42.43, d.f. = 3 [P <0.0001), I^2^ = 93%.

**Unclear risk studies only^2^**

^
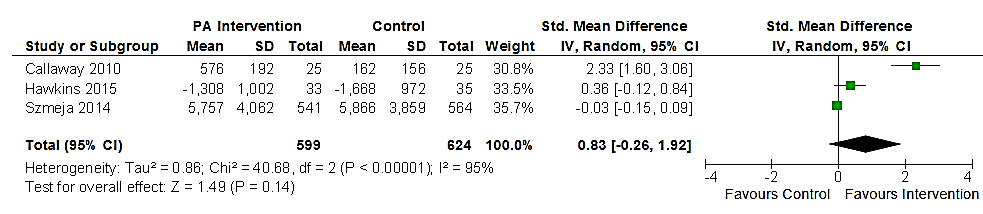
^

^2^ Removing high risk Oostdam et al (2012) and low risk Dodd et al (2014), Kennelly et al (2018), Poston et al (2013) and Van Horn et al (2018)

A meta-analysis for MET minutes per week including unclear risk studies only, demonstrated a non-significant increase in MET minutes per week (SMD 0.83 [-0.26, 1.92], Z = 1.49 P = 0.14). However, the studies were significantly heterogeneous (χ^2^ = 40.68, d.f. = 2 [P <0.0001), I^2^ = 95%.

**Low risk and unclear risk studies^3^**


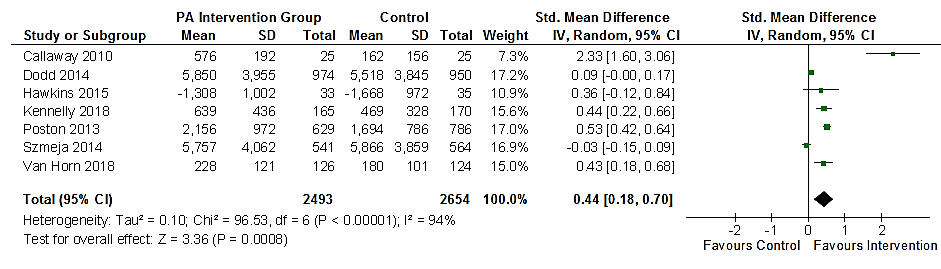


^3^ Removing high risk paper Oostdam et al (2012)

A meta-analysis for MET minutes per week including low and unclear risk studies demonstrated a significant increase in MET minutes per week (SMD 0.44 [0.18, 0.70], Z = 2.67 P = 0.008). However, the studies were significantly heterogeneous (χ^2^ = 96.53, d.f. = 6 [P <0.0001), I^2^ = 94%.

**Steps Count Data**

**Low risk studies only^1^**

^
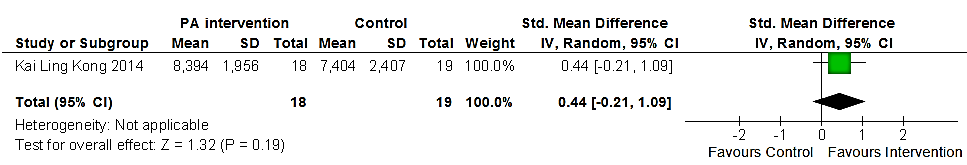
^

^1^ Removing unclear risk paper Bruno et al (2017) and Renault et al (2014)

A meta-analysis for step count including one low risk study only, demonstrated a non-significant increase in steps (SMD 0.44 [-0.21, 1.09], Z = 1.32 P = 0.19) with heterogeneity not applicable.

**Unclear risk studies only^2^**

^
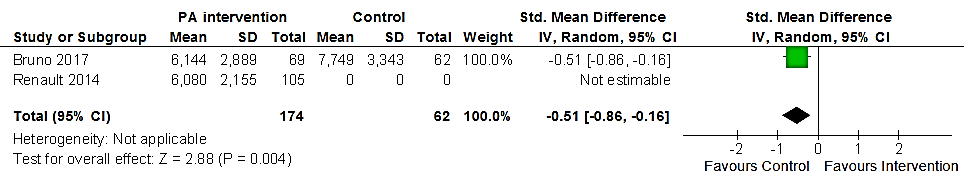
^

^1^ Removing low risk paper Kai Ling Kong et al (2014)

A meta-analysis for step count including unclear risk studies only, demonstrated a significant decrease in steps (SMD -0.51 [-0.86, -0.16], Z = 2.88 P = 0.004) with heterogeneity not applicable.
