# Supplementary material for: Effectiveness of physical activity interventions for overweight and obesity during pregnancy: a systematic review of the content of behaviour change interventions
Source: Int J Behav Nutr Phys Act. 2019 Nov 1;16:97. doi: 10.1186/s12966-019-0859-5 (PMC6825353; doi:10.1186/s12966-019-0859-5)
Supplement: Supplementary file 4 — Additional file 4: Figure S2. Funnel plot. [file 12966_2019_859_MOESM4_ESM.docx]

**Figure S2: Funnel plot for metabolic equilevant (MET)**

**MET m/wk**

**SE (SMD)**

**SE, standard error; SMD, std mean difference**
